# Exposure to Ultrafine Particles from Ambient Air and Oxidative Stress–Induced DNA Damage

**DOI:** 10.1289/ehp.9984

**Published:** 2007-04-27

**Authors:** Elvira Vaclavik Bräuner, Lykke Forchhammer, Peter Møller, Jacob Simonsen, Marianne Glasius, Peter Wåhlin, Ole Raaschou-Nielsen, Steffen Loft

**Affiliations:** 1 Institute of Public Health, Department of Environmental and Occupational Health, University of Copenhagen, Copenhagen, Denmark; 2 Department of Atmospheric Environment, National Environmental Research Institute, Roskilde, Denmark; 3 Institute of Cancer Epidemiology, Danish Cancer Society, Copenhagen, Denmark

**Keywords:** air pollution, biomarkers, Comet assay, DNA repair, oxidative DNA damage, ultrafine particles

## Abstract

**Background:**

Particulate matter, especially ultrafine particles (UFPs), may cause health effects through generation of oxidative stress, with resulting damage to DNA and other macromolecules.

**Objective:**

We investigated oxidative damage to DNA and related repair capacity in peripheral blood mononuclear cells (PBMCs) during controlled exposure to urban air particles with assignment of number concentration (NC) to four size modes with average diameters of 12, 23, 57, and 212 nm.

**Design:**

Twenty-nine healthy adults participated in a randomized, two-factor cross-over study with or without biking exercise for 180 min and with exposure to particles (NC 6169-15362/cm^3^) or filtered air (NC 91-542/cm^3^) for 24 hr.

**Methods:**

The levels of DNA strand breaks (SBs), oxidized purines as formamidopyrimidine DNA glycolase (FPG) sites, and activity of 7,8-dihydro-8-oxoguanine-DNA glycosylase (OGG1) in PBMCs were measured by the Comet assay. mRNA levels of *OGG1,* nucleoside diphosphate linked moiety X-type motif 1 (*NUDT1*), and heme oxygenase-1 (*HO1*) were determined by real-time reverse transcriptase–polymerase chain reaction.

**Results:**

Exposure to UFPs for 6 and 24 hr significantly increased the levels of SBs and FPG sites, with a further insignificant increase after physical exercise. The OGG1 activity and expression of *OGG1, NUDT1,* and *HO1* were unaltered. There was a significant dose–response relationship between NC and DNA damage, with the 57-nm mode as the major contributor to effects. Concomitant exposure to ozone, nitrogen oxides, and carbon monoxide had no influence.

**Conclusion:**

Our results indicate that UFPs, especially the 57-nm soot fraction from vehicle emissions, causes systemic oxidative stress with damage to DNA and no apparent compensatory up-regulation of DNA repair within 24 hr.

Particulate matter (PM) in ambient air is an important risk factor for acute and long-term adverse effects related to pulmonary and cardiovascular diseases, cancer, and mortality (Pope and Dockery 2006). Traffic-related PM may be particularly relevant to these health effects, as indicated by studies on both acute and long-term effects (Hoek et al. 2002; Peters et al. 2004). The ultrafine particle (UFP) fraction of PM with a diameter of < 100 nm typically consists of “fresh” combustion emissions of which vehicle engines are the primary source in urban areas (Sioutas et al. 2005). For UFPs, the size, surface area, chemical composition, and ability to translocate through the epithelium of terminal bronchioles and alveoli are thought to be important in relation to adverse health effects (Delfino et al. 2005; Oberdörster et al. 2005). The mechanisms of action of PM are thought to involve inflammation and oxidative stress, with small particles being more potent than larger particles because of their higher surface area and reactivity (Borm et al. 2004; Knaapen et al. 2004). Experimental studies in animals and cell cultures indicate that DNA can be oxidized by both UFPs and larger (PM_10_; PM with aerodynamic diameter < 10 μm) particle size modes (Knaapen et al. 2004; Risom et al. 2005). DNA damage has been studied mainly as DNA strand breaks (SBs) and guanine oxidation products. The oxidation of guanine studied is primarily 8-oxoguanine, which is mutagenic (Moriya 1993) and related to carcinogenesis (Loft and Møller 2006; Loft et al. 2006). Biomonitoring studies have shown associations between exposure to UFPs, PM_2.5_ (PM with aerodynamic diameter < 2.5 μm), and transition metals (both mass and content) in urban air and the level of oxidized guanine in DNA of peripheral blood mononuclear cells (PBMCs) (Avogbe et al. 2005; Sørensen et al. 2003b, 2005b; Vinzents et al. 2005). However, these studies did not address the time course of DNA oxidation during exposure, identify responsible size modes or sources, or control confounding from other air pollutants, including gases and volatile organic compounds.

Moreover, 8-oxoguanine is removed from DNA by 7,8-dihydro-8-oxoguanine-DNA glycosylase (OGG1), whereas the nucleoside diphosphate linked moiety X-type motif 1 (NUDT1) enzyme removes 8-oxo-2'-deoxy-guanosine 5'-triphosphate (8-oxo-dGTP) from the nucleotide pool, preventing incorporation of 8-oxoguanine during repair processes or replication (Loft and Møller 2006). Experimental studies indicate that acute exposure to PM induces DNA oxidation in target organs, whereas long-term exposure appears to increase the OGG1 repair activity and the oxidative stress response and defense enzyme heme oxygenase-1 (HO-1); this new steady-state situation during continued exposure may be associated with unchanged levels of DNA damage because of increased repair activity (Risom et al. 2003, 2005). If this situation also occurs in human cells, the actual ongoing oxidative stress and detrimental effects of PM may be underestimated by the levels of oxidatively damaged DNA in PBMCs.

Our objective in this study was to use carefully controlled exposure of healthy adults to real-life ambient air particles to delineate the relationship between source-specific particle size modes and oxidation in DNA of PBMCs. Physical exercise was included in the study to mimic real-life exposure because it increases the dose by an increase in the ventilation rate (Daigle et al. 2003), whereas the deposition rates of particles (12–320 nm) may be unaffected by exercise (Löndahl et al. 2007). Chamber air was monitored continuously for size distribution, total particle numbers, and concentration of gases. DNA damage, assessed as SBs and oxidized guanines in PBMCs, and OGG1 repair activity were determined by the comet assay, whereas mRNA expression was measured by real-time reverse transcriptase–polymerase chain reaction (RT-PCR).

## Materials and Methods

### Study population

We invited volunteers to participate by posting a notice in a local newspaper and on campus at the University of Copenhagen. After preliminary screening we recruited 30 nonsmoking, healthy volunteers with no personal history of cardiovascular disease. The sample size was based on prestudy considerations of statistical power. In our earlier studies we detected statistically significant associations between 24- and 48-hr cumulated personal exposure and levels of damaged DNA by repeated measurements in 15–50 volunteers (Sørensen et al. 2003a, 2003b; Vinzents et al. 2005).

Twenty-nine of the 30 volunteers completed the entire program. The participants consisted of 20 men and 9 women, 20–40 years of age (median age, 25 years), with normal lung function (baseline forced expiratory volume in 1 sec: 4.53 ± 0.8 L) and a mean body mass index of 23.0 [95% confidence interval (CI), 22–24]. Participants were taking no medications other than contraception (5 women) and vitamin/mineral supplements (10 participants).

The study was approved by the local ethics committee and in accordance with the Declaration of Helsinki. All participants gave written, informed consent before the study commenced.

### Study design

We used a single blind two-factor cross-over study design with randomized exposure to particles and/or cycling scenarios. Each participant was his/her own control, which excluded confounding by factors that are stable within an individual over time but vary between participants. To avoid a diurnal effect, participants entered the exposure chamber at the same time of the morning on each visit at either 0700 or 0730 hours and stayed for the following 24 hr. The exposure chambers were small offices with a volume of 30 m^3^. Two exposure scenarios were simulated by pumping either nonfiltered air (NFA) with UFP- or particlefiltered air (PFA) into the chambers located on the fifth floor above one of Copenhagen’s busiest roads (Tagensvej). This road consists of three vehicle laneways and a busway. The traffic density on Tagensvej in 2005 was 49,200 vehicles/24 hr (weekdays), including 4–6% heavy duty vehicles (> 3.5 tons) such as buses, lorries, and larger vans (Municipality of Copenhagen 2006). Outdoor air was pumped directly into the chamber using a KVR-100 channel ventilator (Øland A/S, Ballerup, Denmark) (230 m^3^/hr, P = 100 Pa) giving continuous air exchange. A heating device kept the air at room temperature. For the particle-free environment a Camfil FARR HEPA filter (226002A1; Camfil A/S, Stockholm, Sweden) was inserted in-line downstream of the ventilator. Gases including nitrogen oxides [(NO_x_) NO + nitric oxide (NO_2_)], ozone (O_3_), and carbon monoxide (CO) were present in both exposure scenarios and continuously monitored. Each exposure scenario included two episodes of 90-min physical exercise on an ergometer bicycle after an exposure time of 1 and 8 hr, respectively. The intensity was controlled by a heart rate monitor (Polar S720i; Polar Electro ApS, Holte, Denmark) and participants worked at 65–75% of their maximal heart rate defined as 220 beats per minute minus age in years.

All measurements were completed within a 5-month period beginning in February 2005. The median interval between individual exposures for each participant was 12 days. Each volunteer was allowed to leave the chamber to visit the bathroom, kitchen, or for measurement of lung function (not reported here). The median 24-hr period outside the chamber for these visits was 99 min. Data on individual diet throughout the study were obtained from self-administered food frequency questionnaires. Blood was sampled after 6 and 24 hr of exposure. Eight blood samples were lost during the study.

### Peripheral mononuclear blood cell separation

PBMCs were collected and isolated in Vacutainer Cell Preparation Tubes (CPT; Vacutainer Systems, Plymouth, UK) according to the manufacturer’s instructions and frozen at –80°C in a mixture containing 50% fetal bovine serum (FBS; GibcoRBL, Renfrewshire, UK), 40% culture medium (RPMI 1640; GibcoRBL), and 10% dimethylsulfoxide (DMSO).

### DNA damage measured by the Comet assay

The levels of SBs and formamidopyrimidine DNA glycolase (FPG) sites were detected by single cell gel electrophoresis (Comet assay), including incubation with buffer and FPG enzyme for detection of SBs and oxidized purines in PBMCs as previously described (Møller 2005; Vinzents et al. 2005). This assay has been validated in an interlaboratory trial [European Standards Committee on Oxidative DNA damage (ESCODD) 2003]. Coded samples from each participant were analyzed in the same batch along with a quality control PBMC sample. The score of 100 comets per slide with a five-class scoring system (arbitrary score range: 0–400) was translated into lesions per 10^6^ bp by means of a calibration curve based on induction of SBs by X ray, which has a known yield (ESCODD 2003; Møller et al. 2004). We used a conversion factor of 0.0261 Gy equivalents per score and the assumption that a human diploid cell contains 4 × 10^12^ Da DNA, corresponding to 6 × 10^9^ bp.

### Measurement of OGG1 activity

The repair activity of PBMCs was determined as the incision activity of substrate DNA treated with Ro19-8022/white light, which generates 8-oxoguanine (Collins 2004; Collins et al. 2001). We introduced oxidized bases into PBMC substrate nuclei by irradiating cells in phosphate-buffered saline solution with 1 μM Ro 19-8022 (Hoffman-LaRoche, Basel, Switzerland) at 0°C. The cells were washed and resuspended in a mixture containing 50% fetal bovine serum, 40% culture medium, and 10% DMSO, (3 × 10^6^ cells/mL) and frozen at –80°C.

For the preparation of PBMC extracts, the cells were centrifuged (300 × *g*, 5 min, 4°C), and the pellet was resuspended in buffer A (45 mM HEPES, 0.4 M KCl, 1 mM EDTA, 0.1 mM dithiothreitol, 10% glycerol, pH 7.8) at a volume of 20 μL per 10^6^ cells. The resuspended cells were divided in aliquots of 50 μL to which 12 μL 1% Triton X-100 was added. The lysate was centrifuged (700 × *g*, 5 min, 4°C), and the supernatant was mixed with 200 μL buffer B (40 mM HEPES, 0.1 M KCl, 0.5 mM NA_2_EDTA, 0.2 mM bovine serum albumin, pH 8). Approximately 3 × 10^4^ substrate cells were embedded in agarose and lysed as described for the Comet assay. Repair incisions were detected by incubation of the agarose-embedded nuclei with 60 μL PBMC extract or buffer B for 20 min at 37°C. The subsequent alkaline treatment and electrophoresis were identical to the conditions used to determine DNA damage using the Comet assay (Vinzents et al. 2005). The level of repair incisions was obtained as the difference in scores of parallel slides incubated with and without PBMC extract. An assaycontrol (PBMC) was included in each electrophoresis run.

### Expression levels of HO-1, OGG1, and NUDT1 mRNA by real-time RT-PCR

The PBMCs were isolated in Vacutainer tubes and cryopreserved in TRIzol reagent (Invitrogen, A/S, Taastrup, Denmark) at –80°C. On the day of analysis the samples were thawed rapidly, and the RNA was extracted according to the manufacturer’s instructions. Approximately 0.4 μg RNA was used for cDNA synthesis in a reaction volume of 20 μL using the TaqMan GeneAmp RT-PCR kit as recommended by Applied Biosystems (Nærum, Denmark). Quantative PCR reactions were carried out in ABI PRISM 7900HT (Applied Biosystems), using primers and cDNA-specific probes purchased from Applied Biosystems. We used as the reference gene 18S rRNA, which is commercially available as a probe and primer solution (Eukaryotic 18S rRNA Endogenous Control, 4352930E; Applied Biosystems). Below are probes and primers for the genes. Sequence accession ID numbers are from GenBank (http://www.ncbi.nlm.nih.gov/Genbank/; accessed 12 February 2007):

*hHO-1*: forward primer: 5′-CAT GAG GAA CTT TCA GAA GGG C-3′; reverse primer: GAT GTG GTA CAG GGA GGC CAT-3; TaqMan probe: 5′-6-FAM-TGA CCC GAG ACG GCT TCA AGC AGC TG-TAMRA-3′ (NM_002133).

*hOGG1*: forward primer: 5′-AAA TTC CAA GGT GTG CGA CTG-3′; reverse primer: 5′-GCG ATG TTG TTG TTG GAG GA-3′; TaqMan probe: 5′-6-FAM-CAA GAC CCC ATC GAA TGC CTT TTC TCT TT-TAMRA-3′ (U96710).

*hNUDT1*: forward primer: 5′-CAT CGA GGA TGG GGC TAG -3′; reverse primer: CAG AAG ACA TGC ACG TCC ATG A-3′; TaqMan probe: 5′-6-FAM-TCG CCC ACG AAC TCA AAC ACG ATC T-TAMRA-3′ (D16581).

We performed the PCR reactions in triplicate using TaqMan Fast Universal PCR Master Mix (Applied Biosystems) according to the manufacturer’s protocol. For the PCR reaction the following protocol was used: activation of TAQ polymerase for 20 sec at 95°C, followed by a total of 45 temperature cycles for 0.01 sec. at 95°C and 20 sec at 60°C. In each run a standard was included and verified on the efficiency plot, and the variation coefficients of the repeated measurements were 2.98%.

### Size distribution, concentration, and elemental composition of PM

In the exposure chambers, the size distribution and number concentration (NC) of fine particles (6–700 nm) were continuously monitored using a custom-built differential mobility particle sizer (Wåhlin et al. 2001), whereas concentrations of O_3_, NO, NO_2_, and CO were measured continuously using monitors from Teledyne API (Advanced Pollution Instrumentation (Teledyne, San Diego, CA, USA). NFA-cumulated 24-hr particle samples were collected using dichotomous stacked filter units (Luhana et al. 2001) as fine (< 2.5 μm diameter) and coarse fractions (10–2.5 μm diameter). Sampling filters were polycarbonate membrane filters (Nucleopore Costar Corp., Cambridge, MA, USA). We determined particle mass in NFA gravimetrically and elemental composition using proton induced X-ray emission as previously described (Wåhlin et al. 2006). Filter-based measurements were not performed on PFA because of the low particle levels.

Outdoor levels of UFPs and gases were also measured at fixed monitoring stations. The first was located on the roof of the 20-m high H.C. Ørsteds campus building (considered background) at the University of Copenhagen in a park area in the center of Copenhagen, approximately 300 m from Tagensvej. The second was located on the curb of H.C. Andersen’s Boulevard (busy street), which has 60,000 vehicles per workday (Kemp et al. 2006).

We calculated particle aerodynamic surface areas and volumes (V) by integrating with respect to particle NC and using particle diameters *d*. The following integrals were used: area = ∫ π *d*^2^ × dNC and V = 1/6 ∫ π *d*^3^ × dNC. Results of size distribution and NC of particles from 6–700 nm in diameter were fitted using four log-normal modes, NC_12_ (median diameter 11.7 nm, geometric width 1.24), NC_23_ (22.6 nm, 1.48), NC_57_ (57.1 nm, 1.96), and NC_212_ (212 nm, 1.72), where the functional dependence of the modes on *d* was defined by Equation 1:


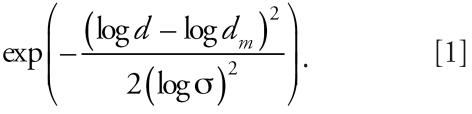


The first three modes represent the characteristic traffic particle modes that have been observed in busy streets in Copenhagen (Wåhlin et al. 2004). The last mode represents secondary long-range transport and a large fraction of particle mass.

Finally, when volunteers were outside the chambers (median, 99 min/24 hr), each carried a Condensed Particle Counter (TSI 3007; TSI Inc., St. Paul, MN, USA). These instruments monitored NC_total_ (10–700 nm), with total exposure concentrations adjusted for these periods.

### Statistical strategy

We investigated the effect of exposure on the outcome variables SBs, FPG sites, mRNA expression of DNA repair genes, and OGG1 repair activity using mixed-effects models by the PROC MIXED procedure of SAS (version 8.2; SAS Institute Inc., Cary, NC, USA). Subject nested within gender was included as a random factor variable to account for interindividual variation. Exposure in terms of presence or absence of particle filter in the air inlet, length of exposure (6 and 24 hr), performance of exercise as well as use of contraceptive pills and consumption of multivitamin supplements were included as categorical explanatory variables. Consumption amounts of fruit and vegetables (grams per day) were included as continuous variables. Subsequently, we investigated dose–response relationships related to exposure in terms of the average NC of particles within each of the four size modes, with mean diameters of 12, 23, 57, and 212 nm. Effects of each of the four size modes were investigated by mutual adjustment with inclusion of all as continuous variables simultaneously in the model. Finally, we estimated effects of exposure adjusted for possible confounding by including length of exposure and exercise as categorical variables and O_3_, CO, NO_x_, and age as continuous variables. The distributions of the exposure concentrations, the DNA damage, and mRNA expression were skewed; therefore all statistical analyses were performed on the natural logarithm of these data, with model estimates representing slopes on the logarithmic scale. Significant differences between concentrations of NC, and gaseous parameters (O_3_, NO, NO_x_, and CO) according to the two exposure scenarios were determined by a *t*-test. In all analyses, *p* < 0.05 was considered to be statistically significant.

## Results

### Exposure characterization

**Figure 1:**
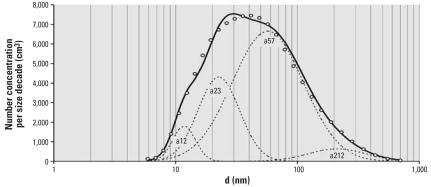
Daily average number concentrations and size distributions of UFPs (6–700 nm in diameter) resolved into four size modes (with median diameters 11.7, 22.6, 57.1, and 212 nm) at an urban background monitoring station in Copenhagen from 15 May 2001–31 December 2004. Abbreviations: a, size mode; d, particle diameter. Vertical lines represent the median diameters on a logarithmic scale. Curved bold line is the measured size distribution and concentration of total particle numbers; dotted lines represent the modeled sum and individual mode (11.7, 22.6, 57.1, and 212 nm) concentration and size distribution.

Table 1 summarizes levels and size distribution of PM and levels of gases during the two different exposure scenarios and during the corresponding period at nearby monitoring stations at a busy street and in an urban background. The 24-hr total NC ranged from 91–542/cm^3^ and 6,169–15,362/cm^3^ for PFA and NFA, respectively. The filter effectively removed particles assessed by all variables (all *p* < 0.01, *t*-test). NO_x_ and NO were unaffected by removal of PM by filtering the air, whereas O_3_ was significantly (*p* < 0.01, *t*-test) reduced (possibly because of a reaction with the filter material) and CO significantly increased (*p* = 0.04, *t*-test). During the NFA scenario the levels of PM and gases in the chambers resembled the composition of urban background air with penetration and mixing with busy street air. The daily 24-hr average of NC_total_ was resolved in four size modes (Figure 1). NC_57_ was the most abundant and also represented the major part of the surface area in both indoor and outdoor (background and urban) air (Table 1). Finally, the chemical composition of air during the NFA exposure scenario (Table 2) shows that the PM_2.5_ fraction was rich in sulfur, which is consistent with substantial contributions from long-range transport. This fraction was also rich in metals and carried relatively high concentrations of transition metals (vanadium, chromium, iron, copper).

### Biomarkers

**Figure 2:**
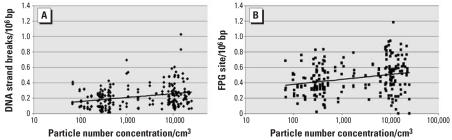
**(***A*) Relationship between SBs and 24-hr average exposure in terms of NC_total_ (6–700 nm). (*B*) Relationship between FPG sites and 24-hr average exposure in terms of NC_total_ (6–700 nm). Individual exposure gradients (NC_NFA_/N_PFA_) were on average 48-fold (range, 2- to 239-fold).

A summary of the levels of DNA damage, OGG1 activity, and mRNA levels according to exposure, exercise, and length of exposure is presented in Table 3. The levels of SBs and FPG sites were significantly increased during NFA exposure compared with PFA exposure independent of the length of the exposure. Exercise had no significant effect, although the exposure-related difference between the median levels of SBs and FPG sites appeared higher during periods of exercise than during periods of rest (Table 3). There were no effects of exposure on the OGG1 activity or mRNA levels of *OGG1*, *NUDT1*, or *HO-1*. The effect estimates in the regression model of the relationships between SB and FPG sites and the exposure variables are presented in Table 4 and this association is shown graphically in Figure 2. The levels of SBs and FPG sites were significantly associated with the NC of all size modes when assessed individually. However, in the regression model, including all size modes, SBs were only significantly associated with NC_57_, whereas FPG sites were significantly associated with NC_23_ and NC_57_. Adjustment for gases, including O_3_, NO_x_, and CO (Table 4), fruit and vegetable intake, or use of multivitamin supplements or contraceptive pills (data not shown) had no significant effects on the predictive value of main exposure variables, which were not significantly associated with any of the biomarkers. There were no significant associations between exposure and either the OGG1 activity (*p* = 0.26) or mRNA expression levels of repair enzymes (*p* > 0.13) including HO-1, OGG1, and NUDT1.

## Discussion

We found that controlled exposure to UFPs, especially the fraction with a median diameter of 57 nm, was associated with oxidative stress in terms of SBs and FPG sites in PBMCs, with possible minor effects of exercise during exposure. We found no sign of up-regulation of the oxidative stress response or DNA repair systems.

DNA damage is considered to be an important initial event in carcinogenesis. Moreover, oxidized DNA in PBMCs is an indicator of systemic oxidative stress relevant for cardiovascular and other outcomes (Li et al. 2003; Schins et al. 2004). The FPG enzyme used in this study recognizes mainly oxidized purines, primarily guanine, such as the premutagenic 8-oxoguanine lesion and the ring-opened formamidopyrimi-dine bases (Collins 2004). In a previous panel study of individuals living in Copenhagen, we found associations between oxidative stress in terms of 8-oxoguanine and oxidation products of proteins and lipids, respectively, and personal accumulated 48-hr exposure to PM_2.5_ expressed as mass and soot (Sørensen et al. 2003a, 2003b). In that study, however, traffic-related sources could not be identified and indoor sources, including candle burning and passive smoking, contributed to the total exposure and might also have contributed to the effects on the biomarkers (Sørensen et al. 2005a). In another study we found dose-dependent relationships between individual UFP exposure and FPG sites in PMBCs from participants the morning after exposure to traffic during biking in streets, indicating that ambient levels of air pollutants in Copenhagen are sufficient to induce oxidative stress, although indoor sources contributed significantly to both cumulated individual exposure to UFPs and the damage levels (Vinzents et al. 2005). The levels of exposure in these studies are comparable, but the participants in the present study were continuously exposed to controlled levels of traffic-generated particles throughout 24 hr. The effects of UFP exposure in the urban air of Copenhagen are smaller than those recorded in participants exposed to heavy air pollution; for example, we found markedly higher levels of SBs and FPG sites in PBMCs of people living in Cotonou, Benin, which is heavily air polluted with UFPs and benzene because of the high intensity of traffic, including old vehicles and motor bicycles running on inferior gasoline (Avogbe et al. 2005). The SBs measured by the alkaline Comet assay represent frank breaks, alkaline labile sites, or transient repair breaks; they are normally rapidly repaired but can be regarded as a reliable biomarker of ongoing exposure in biomonitoring studies of genotoxic effects in environmental and occupational settings (Collins 2004; Møller et al. 2000). Exposure to particles has been associated with elevated levels of SBs in cell culture systems and animal experimental models (Risom et al. 2005). In our previous study, we found no effect of biking in busy streets on the level of SBs the following morning, whereas FPG sites were elevated, which may be because of rapid repair of the former (Vinzents et al. 2005). The increased level of SBs in PBMCs of exposed participants in this study is consistent with an effect due to the continuous exposure until the time that blood was sampled. Similarly, high levels of SBs were associated with ongoing exposure to UFPs, benzene, and/or possibly other air pollutants among inhabitants in Benin (Avogbe et al. 2005). We observed no effect of exposure on the expression of the enzymes involved in repair of 8-oxoguanine in DNA and the nucleotide pool or in HO-1. In animals, exposure to diesel exhaust particles through inhalation of 20 mg/m^3^ for 4 days or in the diet for 3 weeks have caused up-regulation of HO-1 and OGG1 in lungs, liver, and colon (Dybdahl et al. 2003; Risom et al. 2003). Apparently, the 24-hr exposure to UFP levels found in urban air is not of sufficient length and/or strength to cause a similar up-regulation of the defense systems in PBMCs despite the presence of significant damage. Thus, the levels of DNA damage are not obscured by changes in repair capacity in the present study.

Exposure chambers have been used previously to study mechanisms of effects of diesel exhaust, wood smoke, and concentrated ambient air particles, but the exposures have been much higher (200–250 μg/m^3^) than in our study (Behndig et al. 2006; Holgate et al. 2003; Lippmann et al. 2005; Mills et al. 2005; Sällsten et al. 2006). None of these studies have measured DNA damage or repair, and size modes of the UFP fraction have not been investigated. Within our exposure chambers we were able to study actual UFP levels encountered in streets with moderate traffic or in dwellings with ventilation to busy streets. The NC_23_- and NC_57_-size modes were associated with oxidative stress effects in terms of DNA damage. The NC_57_-size mode mainly represents carbonaceous soot from diesel engine exhaust and the largest fraction of surface area, whereas the NC_23_-size mode represents condensed semivolatile organic compounds from diesel vehicles. These size modes have high deposition fractions, which for hydrophobic UFPs with diameters of 12–64 nm were found to be above 50% in our participants (Löndahl et al. 2007). Although the extent of translocation of UFPs has been debated, the small aerodynamic diameter (e.g., 20–60 nm) is likely to be required (Kreyling et al. 2006; Wiebert et al. 2006). Particles in this size range readily induce cellular oxidative stress and DNA damage because of their large surface area and reactivity (Borm et al. 2004; Knaapen et al. 2004; Risom et al. 2005). Accordingly, systemic oxidative stress and DNA damage is biologically plausible in relation to these UFPs. Moreover, the consistent association between exposure to UFPs as number or PM_2.5_ mass and guanine oxidation in DNA of PMBCs, seen in the present and previous studies (Sørensen et al. 2003a; Avobge et al. 2005; Vinzents et al. 2005), suggests that this is a highly sensitive biomarker of systemic exposure, even if translocation is marginal. Unchanged repair of oxidized guanine during exposure and very limited effects of diet and multivitamin supplement use or other exposures indicate some specificity for UFPs in studies within individuals, whereas interindividual variation is substantial and specificity does not necessarily extend to cross-sectional studies (Loft and Møller 2006; Møller and Loft 2006).

We were not able to study the chemical composition of the UFP size modes. The PM_2.5_ fraction in the chamber showed high levels of sulfur and transition metals such as iron, chromium, copper, and vanadium, which have been associated with high levels of 8-oxoguanine in PBMCs (Sørensen et al. 2005b). However, sulfur and vanadium may be related to long-range contributions from the 212-nm-size mode, whereas the major part of copper in busy streets is in the form of larger (3 μm) brake-wear particles, with only a minor part from tail pipe emissions (Wåhlin et al. 2006).

Exercise may increase the internal dose of air pollutants because of enhanced ventilation (Daigle et al. 2003). A modest effect of exercise was expected because the deposition fraction of UFPs is not increased during exercise, and the actual increase in ventilation was limited to the two 90-min periods of cycling. The nominal difference between the median values of SBs and FPG sites during exposure to NFA and PFA was higher during exercise than during rest (Table 3), although there was no significant effect of exercise in the mixed-effects model. Accordingly, our data are compatible with an increased systemic dose and oxidative stress due to UFPs during exercise.

The irritant and oxidant gases O_3_ and NO_2_ are usually present in ambient air and may be potential confounders when studying effects of particles (Pereira et al. 2005). The chamber NO_2_ levels were constant, and the relatively low O_3_ levels decreased further during filtration of the inlet air, probably due to reaction with the filter material. None of the gases had any significant associations with the biomarkers and adjustment for their levels had only minor influence on the associations between UFP exposure and the biomarkers of DNA damage.

## Conclusion

Controlled exposure to UFPs, especially related to the NC_57_ mode, was significantly associated with oxidation of guanines and SBs in DNA of PBMCs, indicating systemic oxidative stress, although there was no sign of up-regulation of relevant defence genes. Exercise may have enhanced the effect of exposure, although this failed to reach statistical significance. The data support that UFPs, mainly from diesel vehicles, cause systemic oxidative stress at exposure levels encountered in streets or in dwellings near busy roads.

## Figures and Tables

**Table 1:** Total and size mode allocated number concentrations (NC), surface area, and volume of particles (aerodynamic diameter, 6–700 nm) as well as gases.^a^

	Exposure chamber		Outdoor monitoring stations	
	NFA	PFA	Urban background	Busy urban street
NC_total_ (no./cm^3^)	10,067 (6,169–15,362)	235 (91–542)	6,571 (4,530–9,645)	22,809 (13,499–31,977)
NC_12_ (no./cm^3^)^b^	1,187 (521–1,320)	15 (5–91)	191 (35–484)	1,692 (774–2970)
NC_23_ (no./cm^3^l)^c^	2,891 (1,978–4,356)	89 (25–134)	931 (169–2,090)	7,759 (3,537–13,413)
NC_57_ (no./cm^3^)^d^	6,136 (4,629–8,345)	166 (107–314)	4,516 (3,096–6,848)	10,115 (6,713–14,950)
NC_212_ (no./cm^3^)^e^	226 (121–376)	8 (5–14)	177 (34–467)	296 (104–626)
Area_total_ (μm^2^/cm^3^)	193 (163–308)	7 (4–12)	157 (99–278)	361 (249–541)
Area_12_ (μm^2^/cm^3^)	0.56 (0.25–0.63)	0.01 (0.00–0.04)	0.09 (0.02–0.23)	0.80 (0.37–1.41)
Area_23_ (μm^2^/cm^3^)	6.30 (4.31–9.49)	0.19 (0.05–0.29)	2.03 (0.37–4.55)	16.91 (7.71–29.23)
Area_57_ (μm^2^/cm^3^)	156 (117–212)	4.2 (2.7–8.0)	115 (79–174)	257 (171–380)
Area_212_ (μm^2^/cm^3^)	57 (31–95)	2.0 (1.3–3.6)	45 (9–119)	75 (26–159)
Volume_total_ (μm^3^/cm^3^)	6 (5–11)	0.3 (0.2–0.4)	5 (3–11)	12 (8–19)
Volume_12_ (μm^3^/cm^3^)	0.00 (0.00–0.00)	0.00 (0.00–0.00)	0.00 (0.00–0.00)	0.00 (0.00–0.00)
Volume_23_ (μm^3^/cm^3^)	0.03 (0.02–0.05)	0.00 (0.00–0.00)	0.01 (0.00–0.02)	0.09 (0.04–0.16)
Volume_57_ (μm^3^/cm^3^)	4.6 (3.5–6.3)	0.13 (0.08–0.24)	3.4 (2.3–5.2)	7.6 (5.1–11.26)
Volume_212_ (μm^3^/cm^3^)	4.2 (2.3–7.0)	0.15 (0.09–0.26)	3.3 (0.6–8.7)	5.5 (1.9–11.7)
NO_x_ (ppb)	25.83 (13.01–49.56)	28.03 (14.43–52.56)	11.56 (7.43–18.36)	59.52 (37.94–88.17)
NO (ppb)	3.24 (0.72–14.49)	3.21 (0.72–17.42)	1.22 (0.41–3.05)	—
CO (ppm)	0.35 (0.25–0.49)	0.41 (0.28–0.57)	0.21 (0.17–0.29)	0.55 (0.39–0.76)
O_3_ (ppb)	12.08 (5.68–18.85)	4.29 (1.99–10.49)	30.05 (23.24–35.27)	19.52 (11.88–26.67)

a Values are median (interquartile range) of 24-hr average exposure scenarios and outdoor monitoring data.

b Nucleation mode of vehicle exhaust system sulfur compounds with low vapor pressure and a median aerodynamic diameter of 11.7 nm.

c Nucleation mode of volatile organic compounds with a median aerodynamic diameter of 22.6 nm.

d Size mode with a median aerodynamic diameter of 57.1 nm and found mainly in soot.

e Secondary long-range transported particles with a median aerodynamic diameter of 212 nm.

**Table 2:** Particle mass [median (interquartile range)] and chemical composition (ng/m^3^) of particles in the exposure chamber air without filtering.

Exposure chamber PM mass concentrations	Total mass (μg/m^3^)	Mass concentrations of elements
PM_10–2.5_	12.6 (7.5–15.8)	Ti (6.26), V (0.43), Cr (0.49), Mn (1.43), Fe (88.11), Ni (0.45), Cu (4.91), Zr (0.59), Mo (0.34), K (51.82), Rb (0.17), Ca (205.52), Sr (4.15), Ba (1.85), Al (80.76), Zn (7.52), Ga (0.0), Sn (0.6), Pb (0.88), Si (153.93), S (79.05), As (0.0), Se (0.02), Sb (0.65), Cl (124.59), Br (1.30)
PM_2.5_	9.7 (7.0–11.6)	Ti (3.81), V (4.81), Cr (4.18), Mn (2.08), Fe (129.02), Ni (1.78), Cu (7.43), Zr (0.61), Mo (0.59), K (59.12), Rb (0.18), Ca (124.59), Sr (2.60), Ba (4.50), Al (16.49), Zn (12.16), Ga (0.03), Sn (1.35), Pb (4.01), Si (65.94), S (466.39), As (0.09), Se (0.25), Sb (0.98), Cl (20.94), Br (1.51)

Abbreviations: Al, aluminum; As, arsenic; Ba, barium; Ca, calcium; Cl, chlorine; Cr, chromium; Cu, copper; Fe, iron; Ga, gallium; K, potassium; Mo, molybdenum; Mn, manganese; Ni, nickel; Pb, lead; Rb, rubidium; S, sulfur; Sb, antimony; Se, selenium; Si, silicon; Sn, tin; Sr, strontium; Ti, titanium; V, vanadium; Zn, zinc; Zr, zirconium.

**Table 3:** Median (interquartile range) of DNA damage, repair activity (OGG1), and mRNA levels according to exposure, physical activity, and length of exposure.

	All		Rest		Bicycling		6-hr exposure		24-hr exposure	
Biomarker	NFA^a^	PFA	NFA	PFA	NFA	PFA	NFA	PFA	NFA	PFA
SBs/10^6^ bp^a^	0.24 (0.14–0.35)	0.16 (0.09–0.25)	0.23 (0.13–0.35)	0.17 (0.09–0.24)	0.25 (0.15–0.35)	0.14 (0.08–0.25)	0.24 (0.15–0.36)	0.17 (0.09–0.24)	0.24 (0.13–0.33)	0.15 (0.08–0.23)
FPG/10^6^ bp^b^	0.53 (0.37–0.65)	0.38 (0.31–0.53)	0.52 (0.37–0.7)	0.40 (0.32–0.53)	0.53 (0.40–0.65)	0.37 (0.27–0.53)	0.52 (0.37–0.70)	0.37 (0.30–0.51)	0.53 (0.37–0.63)	0.39 (0.31–0.55)
OGG1 activity (a.u.)^c^	50.1 (36.8–64.1)	47.0 (39.9–60.3)	50.8 (39.3–64.6)	48.1 (38.9–60.4)	47.8 (38.3–61.9)	46.4 (41.5–59.5)	50.8 (39.3–64.6)	46.4 (38.6–58.8)	49.8 (37.0–65.4)	47.9 (42.4–60.6)
OGG1 mRNA (× 10^−6^)^d^	6.0 (1.9–20.1)	5.7 (1.8–25.0)	7.1 (2.1–41)	4.3 (1.8–18)	5.5 (1.9–14)	8.1 (2.6–37)	4.1 (1.8–19)	4.3 (1.7–19)	7.0 (2.4–35)	7.9 (2.4–25)
NUDT1 mRNA (× 10^−5^)^e^	2.14 (1.2–6.6)	2.9 (1.0–7.0)	2.8 (1.3–8.8)	2.9 (0.95–7.3)	2.1 (1.1–4.4)	2.9 (1.3–7.0)	2.0 (1.0–6.6)	3.0 (1.3–9.4)	2.5 (1.3–7.4)	2.7 (1.0–6.4)
HO–1 mRNA (× 10^−7^)^f^	7.35 (4–24)	10.9 (4.5–33)	6.3 (4.7–28)	9.5 (4.7–33)	7.5 (4.5–18)	11 (4–44)	7.5 (4.5–24)	9.4 (4.5–31)	6.5 (3.6–18)	12 (4.2–36)

a DNA strand breaks.

b Oxidized purines as formamidopyrimidine DNA glycosylase sites

c Repair incision (arbitrary units).

d mRNA expression of *OGG1*.

e mRNA expression of *NUDT1.*

f mRNA expression of *HO-1*

**Table 4:** Effect estimates of the relationship between SB and FPG and exposure variables expressed as categorical and size mode allocated continuous 24-hr average NC_12_,NC_23_,NC_57_, and NC_212_^a^

	Single-size mode exposure model		Single-size mode exposure model with adjustment for gases		Multiple-size mode exposure model with mutual adjustment and adjustment for gases	
Outcome variable, exposure variable	Estimates (95% CI)	% increase	Estimates (95% CI)	% increase	Estimates (95% CI)	% increase
DNA SBs/10^6^ bp						
Categorical	0.459 (0.34–0.58)^b^	—	0.580 (0.41–0.75)^b^	—	—	—
NC_12_	0.080 (0.05–0.11)^b^	5.7	0.082 (0.04–0.12)^b^	5.8	0.055 (–0.02–0.14)	3.8
NC_23_	0.091 (0.06–0.12)^b^	6.5	0.079 (0.03–0.12)^b^	5.6	–0.101 (–0.21–0.00)	–6.7
NC_57_	0.119 (0.09–0.15)^b^	8.6	0.134 (0.09–0.18)^b^	9.7	0.126 (0.04–0.22)^b^	9.1
NC_212_	0.102 (0.07–0.14)^b^	7.3	0.109 (0.06–0.16)^b^	7.8	0.029 (–0.06–0.11)	2.0
FPG sites/10^6^ bp						
Categorical	0.267 (0.17–0.36)^b^	—	0.221 (0.09–0.35)^b^	—	—	—
NC_12_	0.045 (0.03–0.06)^b^	3.1	0.040 (0.01–0.07)^b^	2.8	–0.033 (–0.07–0.01)	–2.3
NC_23_	0.066 (0.04–0.09)*b*	4.7	0.054 (0.02–0.09)^b^	3.8	0.066 (0.01–0.13)^b^	4.7
NC_57_	0.070 (0.04–0.09)^b^	5.0	0.055 (0.02–0.09)^b^	3.8	0.040 (0.00–0.09)^b^	2.8
NC_212_	0.065 (0.04–0.09)^b^	4.6	0.047 (0.01–0.08)^b^	3.3	excluded (*p* = 0.98)	—

CI, 95% confidence interval.

a We used mixed model regression regarding subject nested in gender as random factor. All model estimates in Table 4 are adjusted for age, exercise, and time of sampling. These three parameters were not significant predictors of SBs or FPG sites in any of the models. The natural logarithms of outcome variables were included and the predictive value (% increase) of estimates is expressed per doubling in exposure variable. Adjustment for gases included O_3_, NO_x_, and CO as the natural logarithm of the average gas concentration, which were not significant predictors per se in any case.

b Statistically significant (*p* < 0.05).
